# Induction and Resuscitation of the Viable but Non-culturable (VBNC) State in *Acidovorax citrulli*, the Causal Agent of Bacterial Fruit Blotch of Cucurbitaceous Crops

**DOI:** 10.3389/fmicb.2019.01081

**Published:** 2019-05-15

**Authors:** Yumin Kan, Na Jiang, Xin Xu, Qingyang Lyu, Vinoj Gopalakrishnan, Ronald Walcott, Saul Burdman, Jianqiang Li, Laixin Luo

**Affiliations:** ^1^Department of Plant Pathology, China Agricultural University, Beijing Key Laboratory of Seed Disease Testing and Control, Beijing, China; ^2^Department of Plant Pathology and Microbiology, The Robert H. Smith Faculty of Agriculture, Food and Environment, The Hebrew University of Jerusalem, Rehovot, Israel; ^3^Department of Plant Pathology, University of Georgia, Athens, GA, United States

**Keywords:** *Acidovorax citrulli*, viable but non-culturable, copper sulfate, induction, resuscitation, pathogenicity

## Abstract

*Acidovorax citrulli* is a gram-negative bacterium that infects a wide range of cucurbits causing bacterial fruit blotch (BFB) disease. Copper-based compounds are the most widely-used chemicals for managing BFB and other bacterial diseases in the field. Many bacteria can enter a viable but non-culturable (VBNC) state in response to stress, including exposure to copper, and recover the culturability when favorable conditions return. The present study demonstrates that *A. citrulli* strain AAC00-1 is able to enter into the VBNC state by treatment with different concentrations of copper sulfate. It took 3 h, 5 and 15 days for all viable cells to lose culturability upon exposure to copper sulfate concentrations of 50, 10, and 5 μM, respectively. The VBNC *A. citrulli* cells regained culturability when the Cu^2+^ ions were removed by chelation with EDTA or by transfer of cells to LB broth, a cell-free supernatant from a suspension of AAC00-1, oligotrophic media amended with casein hydrolysate or watermelon seedling juice. We also found that the VBNC cells induced by Cu^2+^ were unable to colonize or infect watermelon seedlings directly, but the resuscitated cells recovered full virulence equivalent to untreated bacterial cells in the log phase. To the best of our knowledge, this is the first report on the VBNC state in *A. citrulli* and the factors that facilitate resuscitation and restoration of pathogenicity.

## Introduction

Bacterial fruit blotch (BFB) is a devastating disease of cucurbits that has caused significant economic losses worldwide since the late 1980s (Somodi et al., 1991; Bahar et al., 2009; Burdman and Walcott, 2012). The causal agent of BFB is *Acidovorax citrulli*, a seed borne gram-negative bacterium that can infect most cucurbitaceous crops, but is particularly damaging to watermelon and melon (Bahar and Burdman, 2010; Burdman and Walcott, 2012). At least two genetically and physiologically distinct groups are reported in *A. citrulli*: while group I strains have been mainly isolated from non-watermelon hosts, and mainly melon, group II strains have been mostly isolated from watermelon (Walcott et al., 2004; Burdman and Walcott, 2012). Utilization of resistant varieties is the most effective strategy to control the disease. However, to date, there are no resistant varieties of cucurbits resistant to *A. citrulli* (Zhao and Walcott, 2018). Although applications of copper-based bactericides, agricultural antibiotics and other protective chemicals can reduce BFB epidemic development after symptoms appear in the field, their efficiency is limited, and the principal method for BFB management is to reduce primary inoculum by excluding *A. citrulli*-contaminated seeds and seedlings (Hopkins, 1991; Hopkins et al., 2003; Wang et al., 2009). Consequently, both seed testing and antimicrobial seed treatments are critical to prevent BFB epidemics. The traditional agar plate method utilizing semi-selective medium in conjunction with species-specific PCR is still considered as the “gold standard” for detection of seed borne plant–pathogenic bacteria, including *A. citrulli*, but this approach does not account for cells in the viable but non-culturable (VBNC) state, as VBNC cells do not produce colonies on standard media (Jiang et al., 2016).

The VBNC state was first described in *Escherichia coli* and *Vibrio cholerae* in 1982 (Xu et al., 1982). Till now, the VBNC state has been reported in 96 bacterial species in response to a range of environmental stresses (Li et al., 2014; Pinto et al., 2015; Highmore et al., 2018). Determining the number of VBNC cells in a mixed sample containing viable, VBNC and dead cells, is not straightforward and involves subtracting the number of culturable cells from the number of viable cells. The number of culturable cells is usually quantified by plating samples on agar media, while viable cells are counted directly using fluorescent dyes to facilitate visualization or via DNA analysis (Fernandes et al., 2014; Tian et al., 2016). The direct viable count (DVC) method utilizes acridine orange combined with nalidixic acid, or the fluorescent dye 5-cyano-2, 3-ditolyl tetrazolium chloride (CTC) to detect cell division or the metabolism of viable bacteria, respectively (Kogure et al., 1979; Proctor and Souza, 2001). On the other hand, the DNA-based assay involves PCR in combination with staining with ethidium monoazide (EMA) or propidium monoazide (PMA). These dyes can only penetrate dead cells that have compromised membranes, to bind DNA and halt PCR amplification (Luo et al., 2008; Zhong et al., 2016; Han et al., 2018). At present, the LIVE/DEAD^^®^^ *Bac*Light^TM^ Bacterial Viability Kit is more commonly used to enumerate viable cells. This method exploits two nucleic acid binding fluorescent dyes that have different permeability across bacterial membranes (Jiang et al., 2016). The first, SYTO9, is a green fluorochrome that can penetrate all bacterial membranes, whilst the second, propidium iodide (PI), is a red fluorochrome that can only penetrate damaged membranes (Berney et al., 2007; Fernandes et al., 2014). Stained cells can be easily distinguished using the optimum excitation wavelengths of the dyes and epifluorescence microscopy or flow cytometry.

The VBNC state can be induced under many conditions, including heavy metal stress, nutrient starvation, abnormal temperatures, oxygen stress and elevated or reduced osmotic potential (Li et al., 2014; Navarrete and Fuente, 2014; Jiang et al., 2016). Till now, the VBNC state has been reported for few plant–pathogenic bacteria, including *Agrobacterium tumefaciens* (Alexander et al., 1999), *Xanthomonas campestris* pv. *campestris* (Ghezzi and Steck, 1999), *X. axonopodis* pv. *citri* (Campo et al., 2009), *Ralstonia solanacearum* (Grey and Steck, 2001; van Overbeek et al., 2004), *Erwinia amylovora* (Ordax et al., 2006, 2009; Santander et al., 2012) and *Clavibacter michiganensis* subsp. *michiganensis* (Jiang et al., 2016). For each of these species the VBNC state can be induced by exposure to copper sulfate. The VBNC state can also be induced by zinc sulfate for *Xylella fastidiosa* (Navarrete and Fuente, 2014) and acetosyringone for *Pseudomonas syringae* pv. *syringae* and *P. s.* pv. *tabaci* (Mock et al., 2015). In addition, Grey and Steck (2001) noted that the VBNC state occurred naturally during the disease cycle of *R. solanacearum*, which could be an important factor in the epidemiology of this pathogen, since VBNC cells can be resuscitated and regain full virulence under favorable conditions.

The resuscitation of VBNC cells can be influenced by a range of factors (Ordax et al., 2006; Pinto et al., 2015). These include removing the stress inducing condition, e.g., by adding a chelating agent, such as EDTA, to copper-stressed cells; or increasing the temperature to resuscitate cells induced by low temperature (Ordax et al., 2006; Imazaki and Nakaho, 2009). In addition, nutrient-rich media and the addition of specific amino acids can elicit the resuscitation of VBNC cells (Özkanca et al., 2009; Pinto et al., 2011). Furthermore, VBNC cells can resuscitate spontaneously *in vivo*. For example, VBNC cells of *Vibrio* spp. could be resuscitated when inoculated into mice (Baffone et al., 2003), and VBNC cells of *Legionella pneumophila* were resuscitated when introduced into amoeba (Alleron et al., 2008). Similarly, the VBNC cells of plant–pathogenic bacteria can be resuscitated *in vivo*. For instance, VBNC cells of *E. amylovora* were resuscitated in King’s B (KB) broth, but also in immature fruits and pear plantlets, with the pear plantlets being the most effective method of resuscitation (Santander et al., 2012). VBNC cells of *C. michiganensis* subsp. *michiganensis* were also isolated from inoculated tomato plants (Jiang et al., 2016).

Given that copper-containing bactericides are widely used for BFB control and copper stress was shown to induce the VBNC state in other bacteria, it is essential to determine whether copper can induce the VBNC state in *A. citrulli* and if so, whether the VBNC state cells play a role in the BFB disease cycle. Therefore, the objectives of the current study were: (1) to develop a reliable method for the detection of the VBNC state in *A. citrulli* cells; (2) to determine the concentration of copper sulfate required to induce the VBNC state in the group II model strain AAC00-1; (3) to investigate resuscitation conditions for VBNC AAC00-1 cells; and (4) to assess the virulence of VBNC and resuscitated cells on watermelon.

## Materials and Methods

### Bacterial Strains and Culture Conditions

*Acidovorax citrulli* strain AAC00-1 was used in this study. The bacterium was routinely cultured on Luria-Bertani (LB) agar for 48 h at 28°C. For preparation of cultures for VBNC induction experiments, single colonies were picked to inoculate 10 mL of LB broth that were incubated at 28°C for 12 h with shaking (120 rpm) until they reached the exponential growth phase, as determined in preliminary growth curve experiments. At this point 2 mL aliquots of the bacterial suspension were transferred to 200 mL of LB broth and incubated for 12 h under the same conditions. The cells were then harvested by centrifugation at 10,610 *g* for 5 min, washed twice with sterilized 0.85% NaCl solution (w/v) to remove traces of the growth media, and resuspended and adjusted to an OD_600_ of 0.5 in sterilized 0.85% NaCl solution. The resulting suspensions were centrifuged and resuspended as described above to prepare a 20-fold concentrated cell suspension containing approximately 10^9^ CFU/mL. These cells were used in the VBNC induction experiments described below.

### Induction and Verification of the VBNC State in *A. citrulli*

The VBNC cells of *A. citrulli* strain AAC00-1 were induced as previously described (Jiang et al., 2016). The maximum non-inhibitory concentration of copper sulfate (CuSO_4_) for the growth of *A. citrulli* in LB broth was determined prior to the induction experiments. Briefly, 100 μL aliquots of 12 h-incubated liquid cultures (∼10^8^ CFU/mL) were inoculated into 10 mL LB broth containing a range of CuSO_4_ concentrations: 0, 0.01, 0.1, 0.5, 1.0, 1.5, 2.0, and 2.5 mM. The cultures were then incubated for 12 h at 28°C with shaking (120 rpm) and the number of cells in each treatment was estimated by plating on LB agar and counting the number of colonies that grew after incubation at 28°C for 48 h. Each treatment consisted of three replicates, and two independent experiments were carried out.

Viable but non-culturable induction was performed using AAC00-1 suspensions generated in 150 mL 1× AB salts solution (1 g L^-1^ NH_4_Cl, 0.3 g L^-1^ MgSO_4_, 0.15 g L^-1^ KCl, 0.01 g L^-1^ CaCl_2_, 2.5 mg L^-1^ FeSO_4_) amended with 0 (negative control), 0.5, 5, 10, or 50 μM CuSO_4_. The final cell concentration in the induction microcosms was approximately 10^7^ CFU/mL. The flasks were incubated at 28°C without shaking for 210 days, during which 3 mL aliquots were collected and evaluated at regular intervals. The number of cells in each sample was counted by flow cytometry (FCM) and by plating on LB agar. When the number of culturable cells in the suspension was less than 0.1 CFU/mL, it was considered to contain no culturable cells and that all viable cells were in the VBNC state (Whitesides and Oliver, 1997; Baffone et al., 2003). For each treatment, there were two independent experiments with three replicates per experiment.

### Enumeration of Total and Viable Cells Using Flow Cytometry

The ratio of viable cells to the total number of cells was assessed using the LIVE/DEAD^^®^^ *Bac*Light^TM^ Bacterial Viability Kit (Invitrogen, Carlsbad, CA, United States), which identifies dead cells according to the integrity of cell membranes. Preliminary experiments indicated that when the two dyes were used simultaneously, a high level of dead cells could dramatically affect the total number of cells detected (those stained by SYTO9). Hence, samples were stained separately to avoid interference. The preliminary experiments also indicated that the recommended concentration of PI, 30 μM (cells:PI, v:v ratio of 1:1), produced sufficient fluorescent intensity in the detection area, but the recommended concentration of SYTO9, 6 μM (cells:SYTO9, v:v ratio of 1:1) produced strong fluorescence beyond the detection area. Therefore different ratios (cells:SYTO9, v:v ratio of 1:1, 2:1, 3:1, and 4:1) were evaluated to determine the optimal concentration of SYTO9, which was found to be 4 μM (cells:SYTO9, v:v ratio of 2:1). Having established the optimal dye concentration, the total number of cells was calculated by comparison with a known number of fluorescent beads in the Trucount tubes (BD Biosciences, San Diego, CA, United States). Twelve-hour-incubated *A. citrulli* cells washed twice with and resuspended in sterilized 0.85% NaCl solution were incubated for 15 min in the dark in the presence of SYTO9 or PI. Excess dye was then removed by washing in 0.85% NaCl, and 800 μL of each sample was transferred to Trucount tubes for analysis. Cells treated with 75% (v/v) ethanol for 10 min were used as the dead cell control. A total of 10,000 events were assessed for each sample using the FACSCalibur flow cytometry system (BD Biosciences, San Diego, CA, United States) with the following settings: amplifier mode set to Log; voltage mode of forward scatter (FSC) set to E00; photomultiplier tube for side scatter (SSC), FL1 for SYTO9 and FL2 for PI, set at 435, 686, and 600 V, respectively; and flow speed set to medium. The number of *A. citrulli* cells in the microcosm was calculated according to the ratio of cells to beads as follows:

$$\text{Concentration of Ac cells (cells/mL)}=\frac{No.\;of\;events\;in\;cell\;region\;\times \;no.\;of\;beads\;per\;tube}{No.\;of\;events\;in\;bead\;region\;\times \;test\;volume}$$

The number of VBNC cells produced during the induction experiments was assessed using 3 mL samples from each induction flask. After washing the cells twice in sterilized 0.85% NaCl solution to eliminate traces of Cu^2+^, 800 μL samples were placed in Trucount tubes for FCM analysis. The cells were then stained using a 2:1 cell:dye ratio (v/v) (667 μL cells and 333 μL SYTO9) for SYTO9 (4 μM) and 1:1 (500 μL cells and 500 μL PI) for PI (30 μM). After incubation in the dark for 15 min, the cells were washed in 0.85% NaCl to eliminate excess dye before 800 μL samples were transferred to 5 mL round-bottom polystyrene tubes (12 mm × 75 mm style, BD Biosciences, San Diego, CA, United States) for analysis by FCM. The remaining bacterial cell suspension (∼1 mL) was then used to prepare a 10-fold serial dilution that was plated on LB agar to assay for culturability. The resulting colonies were counted after 48 h incubation at 28°C. The number of VBNC cells was then calculated by subtracting the number of culturable cells from the number of viable cells, which in turn was calculated by subtracting the number of dead cells stained with PI from the total number of cells stained with SYTO9. Each treatment consisted of three replicates with the entire experiment being conducted twice.

### Resuscitation of VBNC Cells *in vitro*

Different strategies were used to resuscitate VBNC *A. citrulli* cells that were induced by a range of CuSO_4_ concentrations (5, 10, and 50 μM). The first treatment involved the removal of the induction factor (Cu^2+^) by addition of the chelator ethylene diamine tetraacetic acid (EDTA disodium salt). The effect of different concentrations of EDTA on the growth of *A. citrulli* in LB was determined prior to the resuscitation experiments with the same method used to assess the effects of CuSO_4_ (see above). The EDTA concentrations tested were 0 (negative control), 5, 10, 50, 100, and 200 μM. The experiment was carried out twice, with three replicates per treatment per experiment. In the EDTA resuscitation treatment, 10 mL of VBNC suspensions were transferred from the induction flasks to sterilized tubes containing 100 μL of EDTA at different concentrations to produce final concentrations of EDTA at onefold (5, 10, and 50 μM), 1.5-fold (7.5, 15, and 75 μM), or twofold (10, 20, and 100 μM) of the concentrations of Cu^2+^ used during induction. The resuscitation microcosms were incubated at 28°C with shaking (120 rpm), and 100 μL samples were checked every 2 days by plating on LB agar. Other treatments tested for resuscitation were growth in an oligotrophic (M9 minimal medium) or in a rich (LB) medium. In these experiments, the VBNC cells from 10 mL induction microcosms were harvested by centrifugation at 10,610 *g* for 5 min and washed twice with 0.85% NaCl before being resuspended in an equal volume of the growth medium (10 mL). The resuscitation microcosms were then incubated at 28°C with shaking (120 rpm) and samples were checked daily by plating on LB agar. If no culturable cells were recovered after 7 days of incubation, it was assumed that no resuscitation occurred. Another resuscitation approach involved resuspending VBNC cells in cell-free supernatant (CFS) collected from the exponential phase of an AAC00-1 culture or in AB salts amended with casein hydrolysate. The CFS was prepared by centrifuging a liquid culture of AAC00-1 grown in LB broth for 12 h and passing the supernatant through a sterilized 0.2 μm Minisart filter (Sartorius AG, Göttingen, Germany). The VBNC cells from 10 mL induction microcosms were then harvested by centrifugation and washed twice with 0.85% NaCl and resuspended in either 10 mL of the CFS, or 10 mL of an AB salts solution amended with 100 μg/mL casein hydrolysate. The resulting cultures were incubated at 28°C with shaking (120 rpm) and checked every 2 days for culturability by plating on LB agar. The last resuscitation treatment involved transferring VBNC cells to M9 liquid media amended with 10% (v/v) fresh watermelon seedling juice in an attempt to simulate conditions found *in vivo*. The watermelon seedling juice was prepared as follows: 3-week-old watermelon (cv. Ruihong; kindly supplied by the Institute of Vegetables and Flowers Chinese Academy of Agricultural Sciences) seedlings were grinded and centrifuged at 10,610 *g* for 5 min, and the supernatant was transferred and passed through a sterilized 0.2 μm Minisart filter (Sartorius AG, Göttingen, Germany). The samples were incubated at 28°C with shaking (120 rpm) and checked every 2 days for culturability by plating on LB agar. Each EDTA treatment contained three replicates and the other treatments contained six replicates. There were two independent experiments for all the resuscitation experiments.

### PCR Identification of Resuscitated Cells

Fifteen colonies were randomly picked from each treatment of the resuscitated cells and verified by PCR using the *A. citrulli* specific primers SEQID4^m^ (5′-GTCATTACTGAATTTCAACA-3′) and SEQID5 (5′-CCTCCACCAACCAATACGCT-3′) (Schaad et al., 2000). The PCR was conducted with a MyCycler^TM^ Thermal Cycler 580BR (Bio-Rad, Hercules, CA, United States) in 25 μL reaction volumes containing 2.5 μL 10×*Taq* reaction buffer, 2.4 μL dNTPs (2.5 mM each), 1 μL each primer (10 μM stock), 0.25 μL *Taq*DNA polymerase (5 U/μL), and 1 μL of a cell suspension that was previously incubated at 100°C for 10 min. ddH_2_O was added to complete the reaction volume to 25 μL. The thermocycler reaction conditions were as follows: denaturation at 95°C for 5 min, followed by 35 cycles of 95°C for 30 s, 53°C for 30 s and 72°C for 30 s, and a final extension at 72°C for 5 min. The resulting PCR fragments were analyzed by electrophoresis on a 1.5% (w/v) agarose gel, with single 246 bp fragments indicating a positive result.

### Virulence Assays With VBNC and Resuscitated Cells

Virulence assays were carried out by seed-transmission and cotyledon infiltration assays with watermelon (cv. Ruihong). Seed-transmission experiments were carried out for both VBNC cells and resuscitated cells as previously described (Bahar et al., 2009). Briefly, VBNC cells (∼10^7^ cells/mL) in 10 mL induction suspensions were washed twice with 0.85% NaCl to remove the copper sulfate and resuspended with the same volume of 0.85% NaCl solution. Resuscitated cells were prepared by picking single 48-h-old colonies from LB plates and inoculating them into 10 mL LB broth. After incubation of 12 h at 28°C with shaking (120 rpm), the suspensions were adjusted to an OD_600_ of 0.5, corresponding to ∼10^8^ CFU/mL. The resulting suspensions were diluted to 10^6^ CFU/mL with sterilized 0.85% NaCl solution and used as inoculum in seed transmission assays. Twenty-four watermelon seeds were incubated for 2 h at 28°C with shaking (120 rpm) in the bacterial suspensions (VBNC cells at ∼10^7^ cells/mL and resuscitated cells at 10^6^ CFU/mL). The inoculated seeds were then dried on sterilized filter paper for 12 h at 28°C before being sown in pots (six seeds per pot) filled with a soil mixture containing perlite, vermiculite and chicken manure at a ratio of 4:4:1 (w/w/w). The pots were kept in a greenhouse (20–28°C) with a 14 h photoperiod for 14 days, after which the fresh shoot weight of each plant was measured and the mean weight value of the seedlings was used to evaluate the virulence among different treatments of resuscitated cells. Non-VBNC induced *A. citrulli* cells that were collected at the exponential phase (growth in LB broth for 12 h) were used as positive control for disease induction, while treatment of seeds with a 0.85% NaCl solution was used as negative control. Two independent experiments were carried out with a total of 34 to 45 replicates (plants) per treatment.

Cotyledon infiltration experiments were carried out with a 100-fold concentrated suspension of VBNC *A. citrulli* cells. For these assays, cells from 100 mL VBNC induced suspension were harvested by centrifugation at 10,610 *g* for 5 min, washed twice with 0.85% NaCl solution to remove the Cu^2+^ ions and then resuspended with 1 mL 0.85% NaCl solution. Fourteen-day-old watermelon seedlings were inoculated by injecting 25 μL of a 100-fold concentrated suspension of induced VBNC *A. citrulli* cells (∼10^9^ cells/mL) into the back of the cotyledons. The plants were incubated in the greenhouse and leaf samples were collected at 7 and 14 days post-inoculation (dpi) for bacterial isolation. Leaf samples were surface sterilized by immersion in a 1% (v/v) NaOCl solution for 1 min and washed in sterilized distilled water three times before being dried on sterilized filter paper. The dried leaves were then ground for 1 min at 30 Hz/s with an automated grinder (Retsch GmbH, Haan, Germany), and homogenized in 1 mL sterilized 0.85% NaCl solution by vortexing. The resulting suspension was streaked onto LB agar and candidate colonies that developed after 48 h incubation at 28°C were checked by PCR using the SEQID4^m^/SEQID5 primer set as described above. Two independent experiments were carried out with 6 replicates (cotyledons) per treatment in each experiment.

### Statistical Analysis

Data were analyzed using the SPSS 19.0 software (IBM, Armonk, NY, United States). Statistical significance (*p* < 0.05) was determined using the Student’s *t*-test, one-way or two-way analysis of variance (ANOVA) and Tukey’s honest significant difference (HSD). The chi-squared test (*p* < 0.05) was used to compare the resuscitation frequencies of different induction conditions and different resuscitation methods.

## Results

### Optimization of Detection of *A. citrulli* Viable Cells by FCM

**FIGURE 1:**
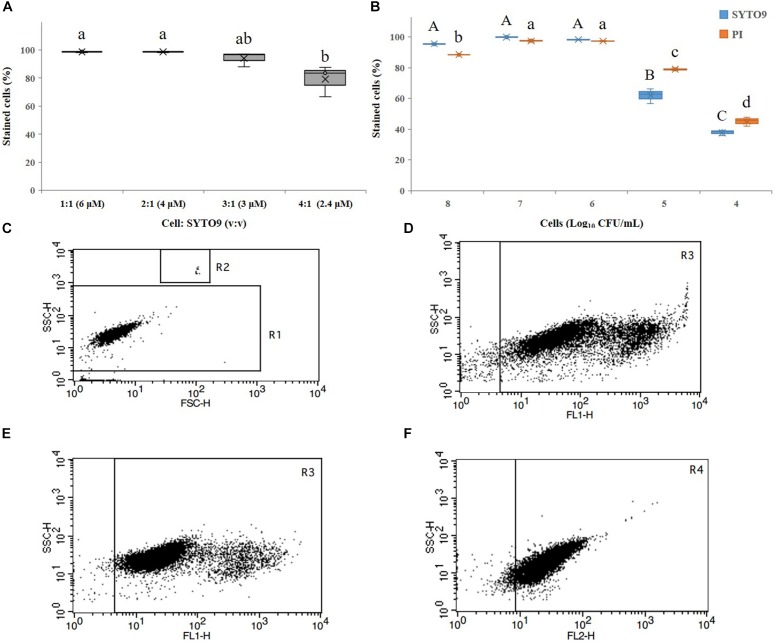
Detection of *Acidovorax citrulli* strain AAC00-1 cells by flow cytometry (FCM): **(A)** Proportion of stained cells resulting from exposure to different concentrations of SYTO9 and different cell: SYTO9 ratios. **(B)** Proportion of propidium iodide (PI)- and SYTO9-stained *A. citrulli* cells resulting from suspensions with different concentrations of *A. citrulli*. **(C)** FCM results produced using *A. citrulli* cells in the exponential growth phase. The bacterial cells are clustered in region R1 and the fluorescent beads are clustered in R2. **(D)** FCM results showing living cells stained with SYTO9 (cell:SYTO9 = 1:1, v:v) clustered in region R3. **(E)** FCM results showing living cells stained with SYTO9 (cell:SYTO9 = 2:1, v:v) clustered in region R3. **(F)** FCM results showing dead cells stained by PI (cell:PI = 1:1, v:v) clustered in region R4. In **(A,B)**, columns and bars show the mean and standard deviation from two independent experiments, each one with three replicates per treatment. Different letters above the bars indicate statistically significant differences according to ANOVA and Tukey’s HSD (*p* < 0.05). In **(B)**, capital letters refer to differences between the SYTO9 treatments while small letters refer to differences between the PI treatments. In **(C–F)**, two independent experiments were carried out with three replicates per experiment having the similar results.

The LIVE/DEAD^^®^^ *Bac*Light^TM^ Bacterial Viability Kit effectively stained cells with a cells:SYTO9 ratio (v:v) of 1:1 and 2:1 (corresponding to final concentration of SYTO9 of 6 and 4 μM, respectively, according to the manufacturer’s instructions) (Figure 1A). However, when the cells:SYTO9 ratio (v/v) was 1:1 and the concentration of SYTO9 was 6 μM, it produced strong fluorescence beyond the detection area (Figure 1D), so a ratio of 2:1 (cells:SYTO9, v:v) was chosen in this study (Figure 1E). PI was used as the manufacturer’s instructions. Additionally, *A. citrulli* cell concentrations in the range of 10^6^ to 10^7^ CFU/mL yielded the best detection rates (Figure 1B). However, since the VBNC induction process was assessed over a long period, 10^7^ CFU/mL was selected for subsequent experiments. The preliminary tests also confirmed that FCM was highly specific, easily distinguishing between fluorescent beads and other debris in the Trucount tube (Figure 1C), and that cells stained by SYTO9 or PI could be clearly distinguished from unstained cells (Figure 1E,F).

### Induction of VBNC Cells by CuSO_4_

**FIGURE 2:**
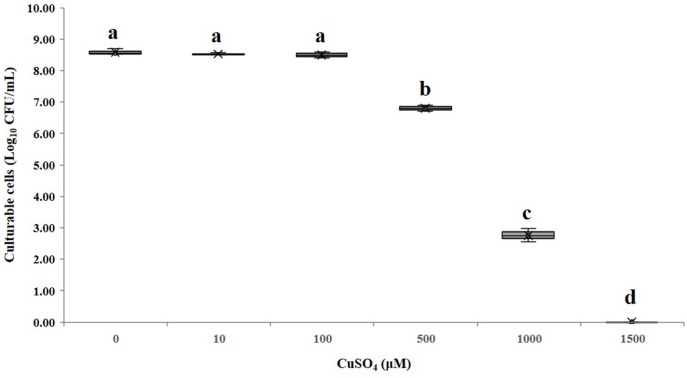
Effect of different CuSO_4_ concentrations on the growth of *A. citrulli* AAC00-1. The bacterial cells were treated with different concentrations of CuSO_4_ for 12 h in LB broth, and then plated on LB agar to estimate concentrations of culturable cells. Columns and bars show the mean and standard deviation from two independent experiments with three replicates per treatment in each experiment. Different letters above the bars indicate statistically significant differences according to ANOVA and Tukey’s HSD (*p* < 0.05).

**FIGURE 3:**
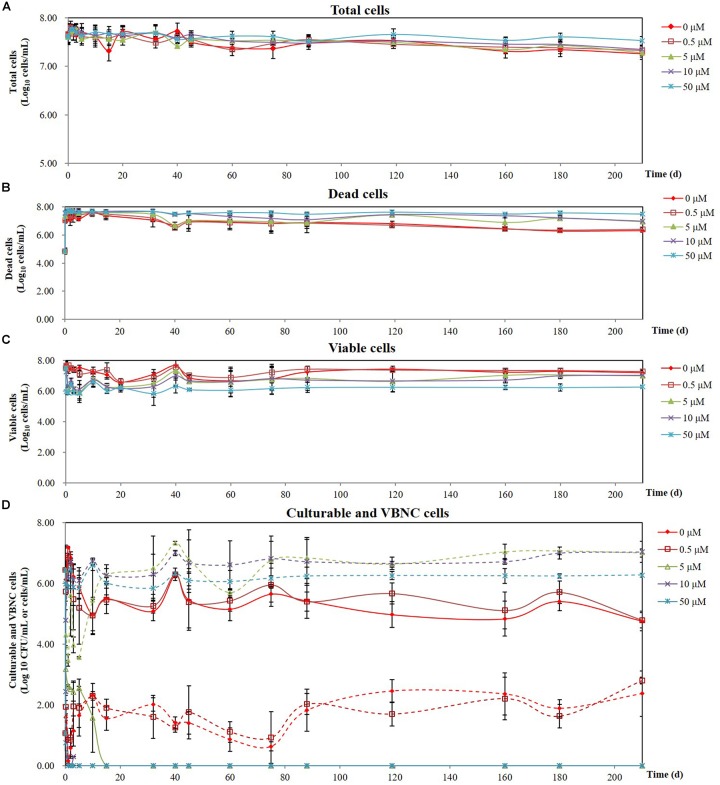
Effect of different CuSO_4_ concentrations on the survival of *A. citrulli* AAC00-1 in AB salts over a period of 7 months: **(A)** total cells; **(B)** dead cells; **(C)** viable cells; **(D)** culturable cells and VBNC cells (solid lines represent culturable cells and dashed lines represent VBNC cells). Concentrations of total cells, dead cells, and viable cells were calculated by flow cytometry using SYTO9 and PI staining, while culturable cells were determined by plating on LB agar. Each data point represents the mean and standard deviation of two independent experiments, with three replicates per treatment in each experiment.

The minimum inhibitory concentration (MIC) of copper sulfate for *A. citrulli* strain AAC00-1 was 1.5 mM, and among tested concentrations, Cu^2+^ concentrations equal and lower than 100 μM had no significant effect on growth in LB broth after 12 h (Figure 2). These results indicated that Cu^2+^ concentrations as high as 50 μM would be suitable for the induction experiments, which were conducted in AB salts solution. The number of total cells, viable cells, culturable cells, and VBNC cells present in the different treatments was assessed over a period of 210 days by combination of FCM and dilution plating methods. The results of these experiments are summarized in Figure 3, while the raw data is detailed in Supplementary Table S1. The highest concentration of copper (50 μM CuSO_4_) induced the VBNC state in a short period of time, with all cells losing culturability after 3 h. The number of viable *A. citrulli* cells decreased from 3.28 × 10^7^ cells/mL to 9.54 × 10^5^ cells/mL. Lower concentrations of copper also induced the VBNC state, although they required longer exposure times: 5 days in the case of 10 μM CuSO_4_ and 15 days at 5 μM, with viable cell counts of 1.24 × 10^6^ cells/mL and 1.89 × 10^6^ cells/mL, respectively. In contrast, the lowest concentration of copper (0.5 μM CuSO_4_) had no effect on culturability compared to the negative control even after 210 days of exposure, with the number of culturable cells being 6.22 × 10^4^ CFU/mL for CuSO_4_-treated cells and 5.79 × 10^4^ CFU/mL for control cells. In spite that the 50 μM treatment had the most rapid effect, with VBNC cell counts of 1.87 × 10^6^ cells/mL, the 10 and 5 μM treatments produced significantly more VBNC cells by the end of the 210 days induction period, with VBNC cell counts of 1.09 × 10^7^ cells/mL and 1.01 × 10^7^ cells/mL, respectively. There were no significant differences between the 10 and 5 μM treatments. All the log-transformed data are shown in Supplementary Table S1 and statistical significance (*p* < 0.05) was according to the Student’s *t*-test (for culturable cells of control and 0.5 μM copper sulfate treatment samples that were exposure 15 days or longer in the copper sulfate) and one-way or two-way ANOVA.

### Resuscitation of VBNC Cells *in vitro*

All resuscitation methods tested were able to induce resuscitation with the exception of the M9 treatment, which failed to resuscitate any VBNC *A. citrulli* cells, regardless of the Cu^2+^ concentration used for VBNC induction (Table 1). EDTA and LB broth were the most effective resuscitation methods. The concentrations of EDTA used for resuscitation had no drastic effect on the growth of *A. citrulli*, although slight but significant differences (*p* < 0.05) were found between 200 μM EDTA and 10 and 50 μM EDTA (Supplementary Figure S1). Resuscitation experiments revealed no significant differences (*p* < 0.05) among 5, 10, and 50 μM induction treatments, and at the three concentrations of EDTA.

**Table 1:** Resuscitation of viable but non-culturable *Acidovorax citrulli* cells induced by CuSO_4_^∗^.

Induction with CuSO_4_	Resuscitation effect (log_10_ bacterial concentration CFU/mL ± SD and resuscitation frequency)									
	EDTA treatment Concentration ratio (EDTA:Cu^2+^)				Nutrient treatment					
		
	1:1	1.5:1	2:1	Statistical analysis for EDTA resuscitation	LB	mM9	CFS	Casein hydrolysate (100 μg/mL)	Seedling juice (10%)	Statistical analysis for nutrient resuscitation
50 μM, 3 h	6.9 ± 0.9 ①	6.7 ± 0.5	7.0 ± 0.2	10/18	7.6 ± 0.6	N ③	8.1 ± 1.4	8.0 ± 0.7	7.9 ± 0.3	19/60
	(2/6) ②	(5/6)	(3/6)	A	(7/12)	(0/12)	(4/12)	(6/12)	(2/12)	a
10 μM, 5 days	6.4 ± 0.3	6.2 ± 0.2	6.8 ± 0.3	11/18	7.5 ± 0.6	N	7.19	6.6 ± 0.07	7.4 ± 0.2	13/60
	(3/6)	(5/6)	(3/6)	A	(7/12)	(0/12)	(1/12)	(3/12)	(2/12)	ab
5 μM, 15 days	5.9 ± 0.2	5.8 ± 0.7	5.6 ± 0.5	14/18	8.4 ± 0.1	N	8.78	N	N	4/60
	(2/6)	(6/6)	(6/6)	A	(3/12)	(0/12)	(1/12)	(0/12)	(0/12)	b
Statistical analysis for all resuscitation treatments	7/18	16/18	12/18	–	17/36	0/36	6/36	9/36	4/36	–
	ABCE	A	AB		ABC	D	CDE	BCE	DE	


① *The average concentration of resuscitated cells and the standard deviation were calculated only from the resuscitated samples;* ② *2/6 means the frequency of resuscitated samples in total tested samples;* ③ *N indicates no culturable cells recovered. ^∗^There were no culturable cells detected in all induced microcosms without resuscitation treatment (data not shown). The chi-squared test was used to evaluate the difference of resuscitation effects caused by induction treatments and resuscitation methods (*p* < 0.05). The results and different letters in 5^*th*^ and last column indicate the statistical analysis for EDTA resuscitation effect (total resuscitation frequency of three concentration ratios of EDTA:Cu^*2*+^) and the statistical analysis for nutrient resuscitation effect (total resuscitation frequency of five nutrient methods) among different induction treatments, respectively. The results and different letters in the last row indicate the statistical analysis for resuscitation effect among the eight resuscitation methods (total resuscitation frequency of three induction conditions).*

As mentioned above, M9 medium treatment was ineffective to induce resuscitation (Table 1). The other tested growth media were more efficient at resuscitating VBNC cells that were exposed to stress conditions for a shorter period of time (e.g., higher CuSO_4_ concentrations). For example, LB broth had resuscitation frequencies of 3/12, 7/12, and 7/12 for the 5, 10, and 50 μM CuSO_4_ induction treatments, respectively, while casein hydrolysate and M9 supplemented with watermelon seedling juice failed to resuscitate VBNC cells from the 5 μM CuSO_4_ treatments. Despite this, when successful resuscitation occurred, the average concentrations of recovered culturable cells were often higher than the concentrations of culturable cells obtained with the EDTA method. For example, resuscitation with LB yielded bacterial concentrations of 2.68 × 10^8^, 3.15 × 10^7^, and 3.81 × 10^7^ CFU/mL for the 5, 10, and 50 μM CuSO_4_ induction treatments respectively. Similarly, resuscitation with CFS of AAC00-1 cells yielded bacterial concentrations of 6.00 × 10^8^, 1.56 × 10^7^, and 1.18 × 10^8^ CFU/mL for VBNC cells induced at 5, 10, and 50 μM CuSO_4_, respectively. Using casein hydrolysate, the average concentrations of resuscitated cells were 3.80 × 10^6^ and 1.07 × 10^8^ CFU/mL, for VBNC cells induced with 10 and 50 μM CuSO_4_, respectively, while for the same VBNC induced cells, M9 supplemented with watermelon seedling juice lead to resuscitation of 2.67 × 10^7^ and 7.77 × 10^7^, respectively (Log-transformed data are shown in Table 1).

### Virulence of VBNC and Resuscitated Cells Toward Watermelon Seedlings

**FIGURE 4:**
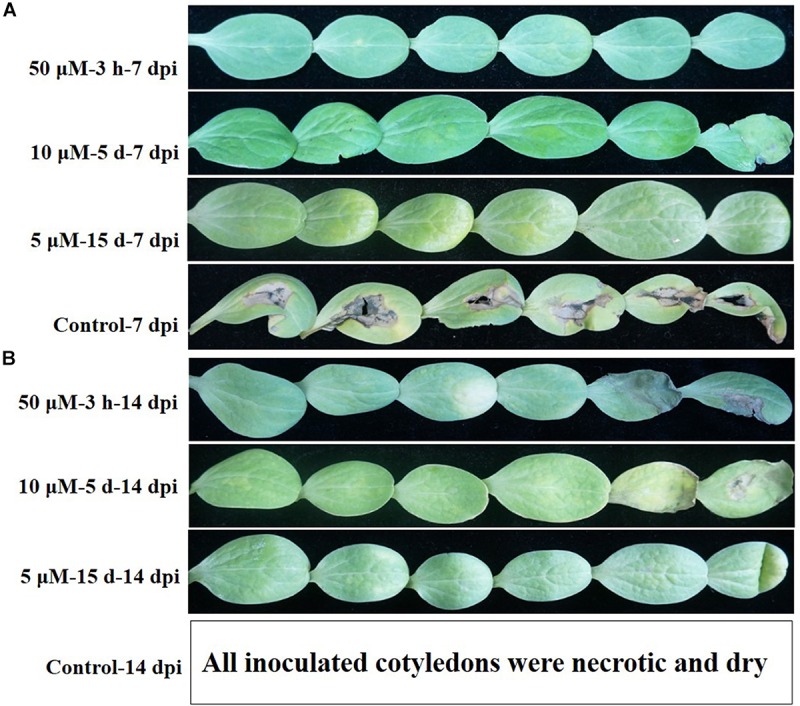
Disease symptoms induced by *A. citrulli* AAC00-1 on watermelon cotyledons at 7 **(A)** and 14 **(B)** days post-inoculation (dpi). Cotyledons were infiltrated with 25 μL VBNC *A. citrulli* cells (∼10^9^ cells/mL) induced by different concentrations of copper sulfate: **(A)** VBNC cells induced by incubation of 50 μM copper sulfate for 3 h, 10 μM copper sulfate for 5 days and 5 μM copper sulfate for 15 days, respectively, and non-induced cells (from line 1 to line 4). **(B)** VBNC cells induced by incubation of 50 μM copper sulfate for 3 h, 10 μM copper sulfate for 5 days and 5 μM copper sulfate for 15 days, respectively (from line 5 to line 7). All cotyledons inoculated with non-induced cells were necrotic and dry at 14 dpi, and we did not take the pictures for this treatment/time (line 8). Six cotyledons were inoculated per treatment and the experiment was repeated twice.

The virulence of the VBNC *A. citrulli* cells produced by exposure to Cu^2+^ was tested by watermelon seed-transmission assays or following injection of 25 μL aliquots of VBNC cells (∼10^9^ cells/mL) into the cotyledons of 14-days-old watermelon seedlings. Watermelon seedlings emerging from seeds that were treated with VBNC *A. citrulli* cells, showed no symptoms in seed-transmission assays (data not shown). Similarly, cotyledons injected with VBNC cells did not develop typical BFB symptoms 14 days post-inoculation (dpi). Furthermore, no culturable *A. citrulli* cells were isolated from any of the cotyledons injected with VBNC cells after this period. In contrast, cotyledons injected with *A. citrulli* AAC00-1 cells that did not undergo through the VBNC state, induced typical water-soaked symptoms at 2 dpi, extensive necrotic lesions at 7 dpi and totally necrotic and dry at 14 dpi (Figure 4).

**FIGURE 5:**
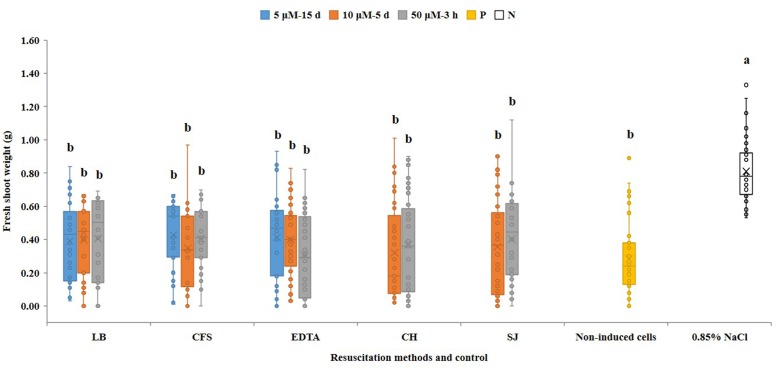
Seed-transmission assays of resuscitated VBNC *A. citrulli* AAC00-1 cells on watermelon. Watermelon seeds (cv. Ruihong) were inoculated with ∼10^6^ CFU/mL bacterial suspensions and let to germinate in pots containing soil mixture in the greenhouse. Treatments were bacterial cells that were induced into the VBNC state with 5, 10 and 50 μM CuSO_4_, and were resuscitated by LB broth (LB), AAC00-1 cell-free supernatant (CFS), EDTA at a concentration of 1.5-fold relative to Cu^2+^ (EDTA), AB salts solution with casein hydrolysate (CH) and M9 minimal medium supplemented with 10% watermelon seedling juice (SJ). Controls were seeds inoculated with AAC00-1 cells that were not exposed to CuSO_4_ (namely non-VBNC cells; non-induced cells) and non-inoculated seeds (seeds treated with 0.85% NaCl). The fresh shoot weight of the seedlings was measured 14 dpi. Columns and bars show the mean and standard deviation from two independent experiments, each one with 34 to 45 replicates per treatment. Different letters above the bars indicate statistically significant differences according to ANOVA and Tukey’s HSD (*p* < 0.05).

In contrast to VBNC cells, resuscitated cells were able to induce symptoms in watermelon seed-transmission assays. In these assays, disease severity negatively correlates with the seedling shoot fresh weight (Bahar et al., 2009). Fourteen days post-inoculation, the mean fresh shoot weight of watermelon seedlings which emerged from seeds inoculated with resuscitated cells from all treatments varied from 0.32 to 0.43 g, and did not significantly differ from seedling emerging from seeds inoculated with non-VBNC treated AAC00-1 cells (0.29 g). All inoculation treatments significantly (*p* < 0.05) differ from non-inoculated controls that were treated with a 0.85% NaCl solution (Figure 5). Taken together these results indicate that VBNC *A. citrulli* cells completely lacked virulence, but once resuscitated, they fully regained pathogenicity ability.

## Discussion

To date 96 different species of bacteria have been reported that can be induced into the VBNC state under laboratory conditions (Li et al., 2014; Pinto et al., 2015; Highmore et al., 2018). This phenomenon is important in plant–pathogenic species since copper-based compounds, which are able to induce VBNC state, are widely used to control bacterial diseases including BFB (Hopkins, 1991; Ordax et al., 2006). Indeed, six of the eight species of plant–pathogenic bacteria investigated in this topic have been shown to enter the VBNC state in response to copper, and four of them can resuscitate under appropriate conditions (Alexander et al., 1999; Ghezzi and Steck, 1999; Grey and Steck, 2001; Ordax et al., 2006; Campo et al., 2009; Jiang et al., 2016).

Our study shows for the first time that the gram-negative bacterium *A. citrulli* can also enter the VBNC state in response to exposure to copper sulfate. Relatively high concentrations of copper sulfate (50 μM) induced the VBNC state of *A. citrulli* cells within hours (3 h), while lower concentrations (5–10 μM) of this compound required many days (5–15 days) for cells to lose culturability. With that said, we observed that the low tested concentrations of copper sulfate produced higher numbers of VBNC cells, possibly because the higher Cu^2+^ concentration resulted in enhanced cell death during the induction process. This study also revealed that there was a lower limit to the effect of copper on induction of the VBNC state, with the 0.5 μM concentration yielding results that were not significantly different to the negative control treatment. These treatments resulted in a low but steady increase in the number of VBNC cells during the 210 days of incubation, which agrees with previous studies that reported that oligotrophic conditions can induce the VBNC state formation and copper can accelerate its induction (Ghezzi and Steck, 1999). In the high concentrations of copper sulfate, the quantity of VBNC cells increased as the culturable cells decreased until there were no culturable cells in the induction microcosm. In this regard, it has been proposed that exposure of VBNC cells to favorable environment conditions may lead to resuscitation; however, if the unfavorable conditions persist, the cells die (Epstein, 2009; Pinto et al., 2015).

Previous reports have described many methods for resuscitation of VBNC cells. For example, EDTA resuscitated VBNC *E. amylovora* cells induced by copper sulfate (Ordax et al., 2006). Similarly, EDTA resuscitated VBNC *A. citrulli* cells at different ratios of EDTA to Cu^2+^. However, the three ratios tested in this study had no significant differences on the rate of resuscitation, and on the average concentration of resuscitated cells. Ordax et al. (2006) found that EDTA can only bind 80% of free copper ions in a solution and that the efficiency of EDTA binding can be further reduced in the presence of the outer membrane of gram-negative bacteria. Importantly, high concentrations of EDTA can be toxic to bacterial cells (Bjerknes and Cheng, 1981; Geider, 1999). However, in our study, we found that EDTA concentrations of up to 200 μM had an effect on *A. citrulli* growth. Overall, these results suggest that EDTA is an effective chelator and useful for resuscitating the VBNC *A*. *citrulli* cells which were induced by copper stress. EDTA is a broad-spectrum metal ion chelator, and whether this compound can induce resuscitation of VBNC bacterial cells which were induced by other heavy metal ions is still to be investigated.

Transfer of VBNC cells to fresh media or addition of specific nutrients have also been used to resuscitate VBNC cells. For example, VBNC *E. amylovora* cells induced by exposure to copper could be resuscitated with King’s B (KB) broth, asparagine or citric acid, but this resuscitation most likely resulted from the chelation of Cu^2+^ ions rather than from nutrients (Ordax et al., 2006). In addition, the VBNC cells can resuscitate *in vivo* leading to infection of the host (Oliver, 2005; Ayrapetyan et al., 2014). VBNC *Vibrio* sp. cells infected mice, and that culturable bacteria could be reisolated from dead mice (Baffone et al., 2003). Similarly, culturable cells of the plant–pathogenic bacteria *E. amylovora* and *C. michiganensis* subsp. *michiganensis* have been recovered from hosts inoculated with VBNC cells (Santander et al., 2012; Jiang et al., 2016). VBNC cells of *R. solanacearum* were able to resuscitate in soil located close to tomato roots (Grey and Steck, 2001). However, not all species of bacteria evaluated could be resuscitated from the VBNC state *in vivo* (Alleron et al., 2013).

We found that VBNC *A. citrulli* cells can be resuscitated by transfer to LB broth but not M9 minimal media. These results are in contrast to those of previous studies that reported that an elevated nutrient environment can inhibit resuscitation in some bacterial species (Whitesides and Oliver, 1997). It has also been suggested that the sudden transfer of VBNC cells from an oligotrophic environment to rich one could inhibit resuscitation due to accumulation of superoxide and free radicals in the bacterial cells (Bloomfield et al., 1998). However, no such effect was found with *A. citrulli* in the current study. It is likely that LB broth provided ideal conditions for resuscitated cells to multiply, while the low nutrient M9 medium could not support bacterial growth.

The resuscitation promoting factor (Rpf), a small protein secreted by *Micrococcus luteus* in the late logarithmic phase of growth, plays an important role in the resuscitation and growth of dormant cells (Mukamolova et al., 1998, 2002; Li et al., 2018). The Rpf protein is widespread amongst bacteria, and has been detected in the supernatants of gram-positive and gram-negative bacterial cultures (Mukamolova et al., 2002; Li et al., 2014, 2018; Ramamurthy et al., 2014). In the public database (NCBI), there are 103 bacterial genes described as “resuscitation promoting factor” (*rpf*). We collected these sequences and compared them by BLAST with the AAC00-1 genome, at nucleotide and amino acid levels. While no homologous genes to *rpf* could be found in AAC00-1 by nucleotide sequence comparison, seven proteins from strain AAC00-1 were found to share some similarity to proteins annotated as Rpf in the public database, though at low levels of identity (data not shown). In the future, it will be interesting to assess if one or some of these proteins act as Rpf in *A. citrulli*. Liu et al. (2009) found that autoinducers in the supernatant of *E. coli* cultures could resuscitate VBNC *E. coli* cells induced by chloraminated or river water. Bacterial CFS can resuscitate VBNC *Vibrio* cells but that the CFS from quorum sensing mutants could not (Ayrapetyan et al., 2014). In our study we found that the CFS derived from exponential phase cultures of AAC00-1 could resuscitate VBNC cells induced by all concentrations of Cu^2+^. However, given that the CFS was sourced from *A. citrulli* cells grown in LB broth, it is unclear whether this induction was due to nutrients remaining in the spent LB broth or by the presence of an Rpf-like or the other quorum sensing signal. In future studies, it will be important to compare the resuscitation inducing ability between CFS obtained from minimal and rich media, and the possible involvement of an Rpf-like or other quorum sensing signal in resuscitation.

There is evidence that the uptake and incorporation of specific amino acids or a combination of different amino acids can stimulate resuscitation of VBNC cells (Pinto et al., 2011; Ramamurthy et al., 2014). We observed that casein hydrolysate can resuscitate VBNC *A. citrulli* cells induced by high concentrations of copper (10 and 50 μM) at low frequency, but interestingly, not in VBNC cells induced by a lower concentration (5 μM) of this ion. Similar results were obtained with M9 minimal media amended with 10% watermelon seedling juice. These results suggest that the resuscitation capacity of VBNC cells might decrease with longer exposure to copper, even at low concentrations of the inducer. Differences in the frequency of success achieved with different resuscitation treatments indicate that the process of resuscitation is complex, probably involving many factors. Furthermore, while there is not a single method that can resuscitate VBNC cells of bacterial species induced by different conditions, VBNC cells induced by a single factor usually can be resuscitated by more than one strategy (Ordax et al., 2006; Zhao et al., 2017). Moreover, a given strategy may lead to different resuscitation results in different bacterial species or even in different strains of the same species (Zhao et al., 2017).

*Acidovorax citrulli* can survive for more than 30 years on or in melon and watermelon seeds and still keep the ability of seed transmission (Block and Shepherd, 2008). It is not clear how the bacterium can survive such a long period in the absence of a growing host. However, clearly, if VBNC cells of plant–pathogenic bacterial species can resuscitate after many years and cause disease, this is a situation with critical implications to disease epidemiology (Baffone et al., 2003; Santander et al., 2012; Jiang et al., 2016). This may not be the case for all pathogens, as the capacity to induce disease depends on the ability of VBNC cells to regain virulence upon resuscitation, and probably on the factors that induced both entrance into the VBNC state and resuscitation. For example, although culturable *C. michiganensis* subsp. *michiganensis* cells could be reisolated from tomato plants inoculated with VBNC cells, the host plants did not exhibit disease symptoms (Jiang et al., 2016). In the present study, we found no evidence that the VBNC *A. citrulli* cells induced by Cu^2+^ are able to resuscitate *in vivo*. With that said, cells that resuscitated *in vitro* exhibited equivalent virulence to that of non-VBNC induced cells. These results suggest that VBNC cells of *A. citrulli*, resulting from exposure to copper-based compounds in the field, could be an important primary inoculum in the BFB disease cycle. Importantly, the concentration of commercial copper-containing bactericides used to control BFB in the field (approximately 45 μM) is similar to the induction condition used in our study (Campo et al., 2009).

In addition to copper chemicals, oligotrophic environment, unsuitable temperatures, antibiotics, drought, and other stress conditions are factors that can induce the VBNC state (Li et al., 2014). Therefore, further research should determine the effects of other stress factors on VBNC state entrance in *A. citrulli*. Additional studies are also needed to implement detection of VBNC *A. citrulli* in the field, for example, in diseased-plant debris, in the soil and in/on seeds, and to determine whether and how VBNC cells of *A. citrulli* resuscitate in the field and the relevance of this phenomenon in the disease cycle. Very little is known about the underlying mechanisms mediating the VBNC state and resuscitation at present (Matilla, 2018), so further studies should be carried out to explore the genetic regulation and the functions of key genes associated to VBNC state.

In the current study, we demonstrated that an *A. citrulli* group II strain AAC00-1 can be induced into the VBNC state by exposure to copper sulfate and that the resulting VBNC cells could be resuscitated and go on to infect host plants. These results infer that the VBNC state cells could be important in the epidemiology of *A. citrulli* and might act as a primary source of BFB inoculum, thus representing a risk for cultivation of cucurbit crops. However, there are at least two genetically and physiologically distinct groups of *A. citrulli* (Burdman and Walcott, 2012; Yang et al., 2019). A previous study showed that group I strains are more resistant to copper than group II strains (Walcott et al., 2004). Therefore, the induction of and resuscitation from the VBNC state of group I strains should be also considered in future studies.

## Data Availability

The raw data supporting the conclusions of this manuscript will be made available by the authors, without undue reservation, to any qualified researcher.

## Author Contributions

This research was primarily conducted by YK, who performed the majority of the experiments, data analysis as well as the preparation of the manuscript. Other authors, including NJ, XX, and QL assisted in the detection of VBNC cells. VG, RW, and SB contributed in discussions and in preparation of the manuscript. JL and LL provided guidance during the experimental design in addition to their critical input in the preparation of the manuscript. All the authors listed above read and approved the final version of the manuscript.

## Conflict of Interest Statement

The authors declare that the research was conducted in the absence of any commercial or financial relationships that could be construed as a potential conflict of interest.
