# Supplementary material for: Induction and Resuscitation of the Viable but Non-culturable (VBNC) State in Acidovorax citrulli, the Causal Agent of Bacterial Fruit Blotch of Cucurbitaceous Crops
Source: Front Microbiol. 2019 May 15;10:1081. doi: 10.3389/fmicb.2019.01081 (PMC6529555; doi:10.3389/fmicb.2019.01081)
Supplement: Supplementary file 1 [file Table_1.pdf]

TABLE S1 | Effect of different $CuSO_4$ concentrations on the survival of *A. citrulli* AAC00-1 in AB salts over a period of seven months: **(A)** Total cells. **(B)** Dead cells. **(C)** Viable cells. **(D)** Culturable cells. **(E)** VBNC cells. Concentrations of total cells, dead cells and viable cells were calculated by flow cytometry using SYTO9 and PI staining, while culturable cells were determined by plating on LB agar.

**(A) Total cells (cells/mL)**					
**Time (d)**	**0 µM** c	**0.5 µM** c	**5 µM** bc	**10 µM** ab	**50 µM** a
0	7.63±0.17	7.63±0.17	7.63±0.17	7.63±0.17	7.63±0.17
0.125	7.69±0.19 A	7.66±0.16 A	7.61±0.11 A	7.62±0.13 A	7.60±0.15 A
1	7.81±0.06 A	7.80±0.07 A	7.70±0.08 A	7.79±0.14 A	7.76±0.08 A
2	7.70±0.16 A	7.68±0.13 A	7.74±0.05 A	7.79±0.04 A	7.76±0.08 A
3	7.64±0.15 A	7.74±0.13 A	7.76±0.10 A	7.71±0.10 A	7.69±0.19 A
5	7.67±0.11 A	7.62±0.09 A	7.56±0.12 A	7.71±0.17 A	7.63±0.17 A
10	7.62±0.14 A	7.59±0.20 A	7.62±0.11 A	7.64±0.21 A	7.70±0.13 A
15	7.32±0.21 B	7.59±0.13 A	7.56±0.13 AB	7.69±0.14 A	7.67±0.08 A
32	7.57±0.05 A	7.49±0.11 A	7.71±0.16 A	7.69±0.16 A	7.68±0.15 A
40	7.72±0.16 A	7.61±0.05 AB	7.42±0.05 B	7.59±0.10 AB	7.55±0.09 AB
45	7.50±0.10 A	7.56±0.10 A	7.55±0.09 A	7.65±0.08 A	7.57±0.14 A
60	7.38±0.07 BC	7.35±0.13 C	7.53±0.05 AB	7.52±0.13 AB	7.62±0.10 A
75	7.36±0.20 B	7.47±0.08 AB	7.54±0.09 AB	7.50±0.06 AB	7.61±0.11 A
88	7.48±0.08 A	7.55±0.11 A	7.52±0.10 A	7.48±0.13 A	7.52±0.11 A
119	7.53±0.11 AB	7.45±0.08 B	7.49±0.03 B	7.52±0.06 AB	7.66±0.12 A
160	7.31±0.14 B	7.40±0.13 AB	7.35±0.08 AB	7.46±0.14 AB	7.53±0.07 A
180	7.34±0.14 B	7.38±0.12 AB	7.43±0.12 B	7.45±0.07 AB	7.60±0.08 A
210	7.26±0.11 B	7.33±0.11 AB	7.28±0.14 B	7.35±0.16 AB	7.53±0.09 A
**(B) Dead cells (cells/mL)**					
**Time (d)**	**0 µM** d	**0.5 µM** d	**5 µM** c	**10 µM** b	**50 µM** a
0	4.84±0.22	4.84±0.22	4.84±0.22	4.84±0.22	4.84±0.22
0.125	7.00±0.17 C	7.06±0.14 BC	7.34±0.26 AB	7.52±0.08 A	7.59±0.15 A
1	7.39±0.27 B	7.15±0.10 B	7.71±0.09 A	7.68±0.13 A	7.76±0.06 A
2	7.06±0.37 B	7.35±0.23 B	7.69±0.06 A	7.72±0.04 A	7.71±0.06 A
3	7.23±0.33 C	7.43±0.08 BC	7.74±0.09 A	7.69±0.09 AB	7.68±0.19 AB
5	7.12±0.09 C	7.41±0.09 B	7.55±0.12 AB	7.70±0.16 A	7.62±0.16 AB
10	7.61±0.16 A	7.54±0.23 A	7.64±0.11 A	7.57±0.14 A	7.71±0.09 A
15	7.39±0.32 A	7.47±0.27 A	7.59±0.16 A	7.67±0.14 A	7.63±0.09 A
32	7.06±0.50 B	7.17±0.22 B	7.47±0.20 AB	7.67±0.15 A	7.67±0.15 A
40	6.70±0.20 B	6.51±0.11 B	6.68±0.04 B	7.44±0.10 A	7.51±0.09 A
45	7.00±0.54 A	6.90±0.64 A	7.02±0.59 A	7.51±0.13 A	7.55±0.15 A
60	6.93±0.46 AB	6.87±0.51 B	7.00±0.53 AB	7.31±0.23 AB	7.60±0.10 A

75	6.85±0.71 A	6.80±0.56 A	7.00±0.66 A	7.17±0.38 A	7.59±0.12 A
88	6.89±0.43 A	6.83±0.36 A	6.88±0.72 A	7.10±0.47 A	7.49±0.12 A
119	6.81±0.18 B	6.68±0.15 B	7.43±0.03 A	7.45±0.10 A	7.64±0.12 A
160	6.45±0.07 C	6.42±0.12 C	6.90±0.32 B	7.36±0.15 A	7.50±0.06 A
180	6.29±0.14 C	6.33±0.09 C	7.19±0.12 B	7.21±0.19 B	7.58±0.08 A
210	6.31±0.10 C	6.40±0.10 C	6.95±0.14 B	6.98±0.11 B	7.50±0.09 A

**(C) Viable cells (cells/mL)**					
**Time (d)**	**0 µM** a	**0.5 µM** a	**5 µM** b	**10 µM** b	**50 µM** c
0	7.52±0.24	7.52±0.24	7.52±0.24	7.52±0.24	7.52±0.24
0.125	7.72±0.17 A	7.66±0.14 AB	7.48±0.13 B	7.26±0.05 C	5.98±0.11 D
1	7.33±0.05 B	7.69±0.07 A	6.09±0.16 CD	6.33±0.12 C	5.88±0.05 D
2	7.43±0.16 A	7.51±0.13 A	6.44±0.11 B	6.73±0.08 B	6.47±0.03 B
3	7.37±0.16 A	7.43±0.19 A	6.14±0.50 B	6.15±0.59 B	5.87±0.60 B
5	7.52±0.14 A	7.11±0.32 A	5.90±0.31 B	6.09±0.43 B	5.85±0.33 B
10	7.27±0.26 A	7.25±0.49 AB	6.78±0.22 BC	6.73±0.10 C	6.59±0.17 C
15	7.05±0.21 A	7.37±0.21 A	6.28±0.33 B	6.27±0.21 B	6.01±0.17 B
32	7.11±0.36 A	6.87±0.71 AB	6.50±1.05 AB	6.30±0.66 AB	5.84±0.43 B
40	7.68±0.16 A	7.57±0.05 AB	7.33±0.05 B	7.00±0.09 C	6.31±0.05 D
45	6.86±0.63 A	7.05±0.57 A	6.62±0.97 A	6.67±0.75 A	6.10±0.40 A
60	6.67±0.72 A	6.89±0.39 A	6.60±1.00 A	6.61±0.79 A	6.06±0.37 A
75	6.81±0.37 AB	7.24±0.19 A	6.76±0.79 AB	6.82±0.57 AB	6.17±0.32 B
88	7.25±0.19 A	7.43±0.09 A	6.83±0.68 AB	6.72±0.72 AB	6.24±0.30 B
119	7.43±0.10 A	7.37±0.09 A	6.63±0.09 B	6.66±0.20 B	6.25±0.14 C
160	7.24±0.17 A	7.35±0.13 A	7.03±0.25 AB	6.72±0.09 B	6.25±0.25 C
180	7.30±0.14 AB	7.34±0.13 A	7.07±0.12 BC	7.00±0.20 C	6.24±0.07 D
210	7.20±0.12 A	7.27±0.11 A	7.00±0.14 A	7.04±0.34 A	6.27±0.06 B

**(D) Culturable cells (CFU/mL)**					
**Time (d)**	**0 µM** a	**0.5 µM** a	**5 µM** b	**10 µM** c	**50 µM** c
0	6.44±0.82	6.44±0.82	6.44±0.82	6.44±0.82	6.44±0.82
0.125	6.13±0.72 A	5.73±0.12 A	3.18±0.78 B	2.44±0.94 B	0.00
1	7.19±0.02 A	6.85±0.14 B	2.63±0.04 C	0.30	0.00
2	6.85±0.19	6.58±0.17	2.51	0.00	0.00
3	6.22±0.39 A	5.47±1.00 A	2.43±0.30 B	0.30	0.00
5	5.87±0.39 A	5.20±1.20 A	2.56±0.06 B	0.00	0.00
10	4.96±0.52 A	4.95±0.60 A	1.58±1.13 B	0.00	0.00
15	5.50±0.50	5.47±0.15	0.00	0.00	0.00
32	5.09±0.32	5.26±0.24	0.00	0.00	0.00
40	6.26±0.07	6.30±0.21	0.00	0.00	0.00
45	5.45±0.92	5.39±0.93	0.00	0.00	0.00
60	5.16±0.39	5.43±0.34	0.00	0.00	0.00
75	5.65±0.26	5.95±0.71***	0.00	0.00	0.00
88	5.43±0.58	5.40±0.31	0.00	0.00	0.00
119	4.97±0.43	5.67±0.34	0.00	0.00	0.00
160	4.84±0.57	5.11±0.60	0.00	0.00	0.00

180	5.41±0.30	5.71±0.37	0.00	0.00	0.00
210	4.76±0.32	4.79±0.25	0.00	0.00	0.00

**(E) VBNC cells (cells/mL)**					
**Time (d)**	**0 µM** c	**0.5 µM** c	**5 µM** b	**10 µM** a	**50 µM** b
0	1.08±0.59	1.08±0.59	1.08±0.59	1.08±0.59	1.08±0.59
0.125	1.59±0.72 C	1.93±0.21 C	4.30±0.83 B	4.79±0.90 AB	5.98±0.11 A
1	0.16±0.09 D	0.85±0.07 C	3.46±0.19 B	6.23±0.15 A	5.88±0.05 A
2	0.58±0.34 B	0.93±0.29 B	5.60±1.34 A	6.73±0.08 A	6.47±0.03 A
3	1.15±0.50 C	1.96±0.82 C	3.98±0.26 B	6.10±0.55 A	5.87±0.60 A
5	1.65±0.38 C	1.91±0.94 C	3.56±0.03 B	6.09±0.43 A	5.85±0.33 A
10	2.31±0.39 C	2.30±0.14 C	5.46±1.14 B	6.73±0.10 A	6.59±0.17 A
15	1.55±0.39 C	1.90±0.29 C	6.28±0.33 B	6.27±0.21 A	6.01±0.17 A
32	2.01±0.22 B	1.61±0.70 B	6.50±1.05 A	6.30±0.66 A	5.84±0.43 A
40	1.42±0.18 D	1.27±0.16 D	7.33±0.05 A	7.00±0.09 B	6.31±0.05 C
45	1.41±0.38 B	1.76±0.88 B	6.79±0.98 A	6.67±0.75 A	6.10±0.40 A
60	0.86±0.39 B	1.12±0.34 B	5.70±0.11 A	6.61±0.79 A	6.06±0.37 A
75	0.63±0.15 B	0.93±0.84 B	6.76±0.79 A	6.82±0.57 A	6.17±0.32 A
88	1.82±0.69 B	2.03±0.35 B	6.83±0.68 A	6.72±0.72 A	6.24±0.30 A
119	2.46±0.38 B	1.70±0.39 C	6.63±0.09 A	6.66±0.20 A	6.25±0.14 A
160	2.36±0.70 C	2.21±0.70 C	7.03±0.25 A	6.72±0.09 AB	6.25±0.25 B
180	1.89±0.28 C	1.63±0.38 C	7.07±0.12 A	7.00±0.20 A	6.24±0.07 B
210	2.37±0.75 C	2.80±0.11 C	7.00±0.14 A	7.04±0.34 A	6.27±0.06 B

**Yellow-highlighted cells: at this time point there were no culturable cells in the induction microcosm and all the viable cells were in the VBNC state.*
Grey-highlighted cells: at this time point we did not detect culturable cells or detected very few colonies in only one replicate and those data were not included in the data analysis.
*Statistical significance by using two-way analysis of variance (ANOVA) showed that there is interaction between the concentration of copper sulfate and induction time, and both of the two factors had significant effect on the number of cells ($p < 0.05$); the significance of each time point was determined using one-way ANOVA and Tukey's HSD ($p < 0.05$) or Student's t-test (for culturable cells of control and 0.5 µM copper sulfate treatment samples that were exposure 15 d or longer time to copper sulfate). Small letters refer to differences between the different concentrations of copper sulfate by two-way ANOVA and capital letters refer to differences at each time point between different concentrations of copper sulfate by one-way ANOVA. Student's t-test showed that there is no significant difference between control and 0.5 µM copper sulfate treatment samples except the 75 d samples (marked with ***). Each data represents the mean and standard deviation of two independent experiments, with three replicates per treatment in each experiment.*
