# Supplementary material for: Induction and Resuscitation of the Viable but Non-culturable (VBNC) State in Acidovorax citrulli, the Causal Agent of Bacterial Fruit Blotch of Cucurbitaceous Crops
Source: Front Microbiol. 2019 May 15;10:1081. doi: 10.3389/fmicb.2019.01081 (PMC6529555; doi:10.3389/fmicb.2019.01081)
Supplement: Supplementary file 2 [file Data_Sheet_1.PDF]

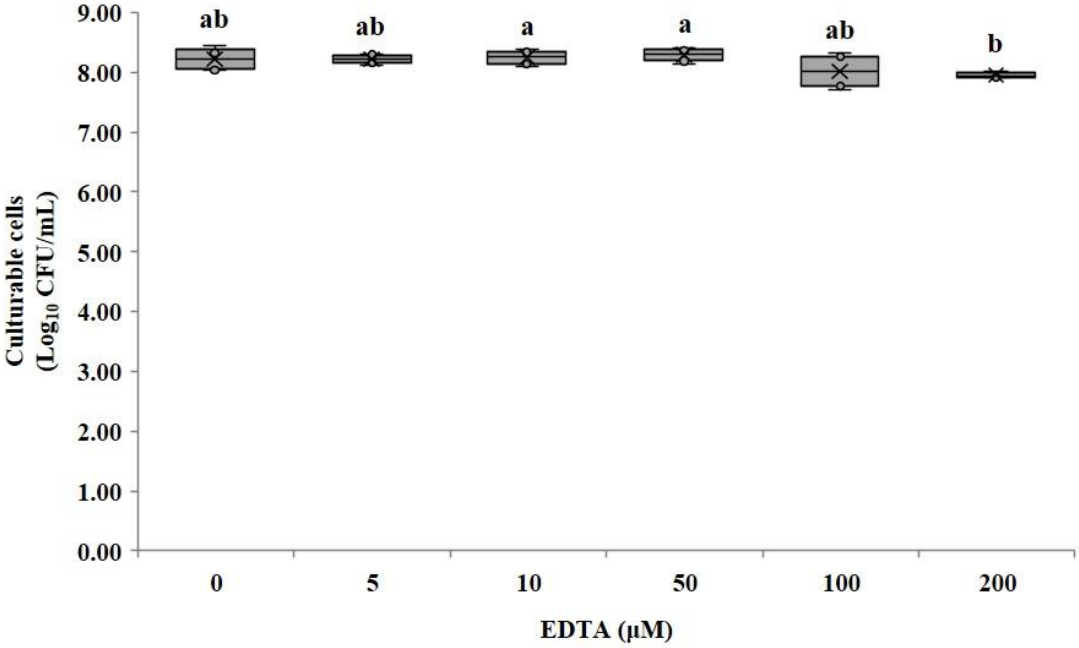

FIGURE S1 | Effect of EDTA concentration on the growth of *Acidovorax citrulli* strain AAC00-1. The bacterial cells were treated with different concentrations of EDTA for 12 h in LB broth, and then plated on LB agar to estimate concentrations of culturable cells. Columns and bars show the mean and standard deviation from two independent experiments and each of them contained three replicates. Different letters above the bars indicate statistically significant differences according to ANOVA and Tukey's HSD ($p < 0.05$).
